# Saccadic intrusions in amyotrophic lateral sclerosis (ALS)

**DOI:** 10.16910/jemr.12.6.8

**Published:** 2019-09-16

**Authors:** Wolfgang Becker, Martin Gorges, Dorothée Lulé, Elmar Pinkhardt, Albert C. Ludolph, Jan Kassubek

**Affiliations:** University of Ulm, Germany

**Keywords:** visual fixation, saccadic intrusions, microsaccades, executive functions, amyotrophic lateral sclerosis, motoneuron disease

## Abstract

The attempt to quietly fixate at a small visual object is continuously interrupted by a variety of fixational eye movements comprising, among others, a continuum of saccadic intrusions (SI) which range in size from microsaccades with amplitudes ≤0.25° to larger refixation saccades of up to about 2°. The size and frequency of SI varies considerably among individuals and is known to increase in neurodegenerative diseases such as progressive supranuclear palsy (PSP), and amyotrophic lateral sclerosis (ALS). However, studies of ALS disagree whether also the frequency of SI increases. We undertook an analysis of SI in 119 ALS patients and 47 age-matched healthy controls whose eye movements during fixation and tests of executive functions (e.g antisaccades) had been recorded by video-oculography according to standardised procedures. SI were categorised according to their spatio-temporal patterns as stair case, back-and-forth and square wave jerks (a subcategory of back-and-forth). The SI of patients and controls were qualitatively similar (same direction preferences, similar differences between patterns), but were enlarged in ALS. Notably however, no increase of SI frequency could be demonstrated. Yet, there were clear correlations with parameters such as eye blink rate or errors in a delayed saccade task that suggest an impairment of inhibitory mechanisms, in keeping with the notion of a frontal dysfunction in ALS. However, it remains unclear how the impairment of inhibitory mechanisms in ALS could selectively increase the amplitude of intrusions without changing their frequency of occurrence.

## Introduction

Frequently used abbreviations marked **bold** on first occurrence

The attempt to steadily fixate at a visual object is continuously interrupted by a variety of fixational eye movements comprising slow drifts (typically ≤ 0.07°/s) and small saccades ranging in amplitude from less than 0.25° ('microsaccades') to about 2°. Early observations of microsaccades were made by Barlow [1], Ditchburn [2], Ratliff [3] and Yarbus [4] who demonstrated saccades as small as 0.02°. Microsaccades may be to some degree spontaneous events but are also thought to offset the effect of drift movements. However they are not necessary for precise fixation because drift can also be controlled in a smooth, non-saccadic way (for a review of this topic see [5]). Other early suggestions related them to the prevention of image fading and to information processing [6, 7], a view supported also by more recent studies [8, 9, 10]. However, other authors, while acknowledging that microsaccades counteract image fading, question whether they are essential for the prevention of fading. [5, 11].

Similar interpretations as above have been advanced concerning the role of the spontaneous saccades of about 0.25 – 2° that repeatedly interrupt fixation, and which have been termed saccadic intrusions, therefore. Microsaccades and saccadic intrusions actually form a continuum: They belong to a common unimodal amplitude distribution and conform to the same peak velocity *vs*. amplitude relationship [12] that applies to nearly all visually guided saccades ('main sequence', [13, 14]). Also, their midbrain and brainstem mechanisms of neural control do not differ from those of larger saccades [15]. Moreover, both types are similarly modulated by attention [16, 17] and can be transiently suppressed by instruction [18]. Therefore, we henceforth will speak indiscriminately of saccadic intrusions (**SI**) whether or not the saccades occurring during fixation are small or large, or whether they are corrective or error-producing.

The characteristics of SI in healthy subjects have been characterised in terms of their frequency of occurrence, their amplitude, their spatial direction and the duration of the saccade-free fixation periods between them. In humans, the majority of SI are horizontally oriented. A characteristic common to the SI of nearly all individuals is their conspicuous dynamic overshoot [19] in the form of an immediate partial return of the eyes which has already been noted by Ditchburn [2]. In extreme cases it can reduce the final steady state eye displacement to less than 50% of the initial peak displacement.

A particular subclass of saccadic intrusions is formed by the so called square wave jerks (**SWJ**) which were first identified in electronystagmographic recordings from neurologic patients [20], originally referred to as* Gegenruck*e [21]. The distinctive hallmark of SWJ is not a particular amplitude range but a spatio-temporal pattern consisting of an initial saccade away from the fixation target followed, after a brief period (200-300 ms), by a saccade back to it. SWJ were originally reported to be rare or absent in healthy subjects [18, 22]. However, with the advent of recording equipment with high spatial resolution, SWJ were found in almost all subjects [23]. The return saccade of SWJ occasionally overshoots the target position so that a third saccade is required to return the eyes to the target, thus creating a 'biphasic SWJ' [19, 24].

A high incidence of SI has been reported in a number of neurodegenerative diseases [25]. Several studies have compared patients with neurodegenerative Parkinsonism including Parkinson's disease, progressive supranuclear palsy (**PSP**) and multi-system atrophy (**MSA**) to healthy controls in terms of various SI parameters. The most consistent result across studies was a significant increase of SI amplitude and SI frequency in PSP [26-28] and MSA [26, 28].

An altered SI activity has also been found in amyotrophic lateral sclerosis (**ALS**) [ 29, 30]. ALS is the most frequent adult-onset motor neurone disease. It is characterised by progressive paresis and leads to death from respiratory failure within about 3 years from disease onset on average [31]. The pathological process underlying ALS is histologically characterised by aggregates of the DNA-binding protein pTDP-43. According to Braak et al. [32] these aggregates typically propagate in a four-stage sequence. Initially, lesions develop in the primary motor cortex and frontal supplementary motor areas and cause muscle wasting in the extremities (stage 1). In stage 2, lesions appear, amongst other areas, in the prefrontal cortex and in the precerebellar nuclei. In stages 3 and 4, the pathology progresses further into the prefrontal cortex and appears also in the striatal area of the basal ganglia and other subcortical structures. Frontal and prefrontal areas control many aspects of eye movements. This control is mostly exerted by via indirect pathways to the eye movement generating structures of the brain stem; the striatal area and the precerebellar nuclei are part of these pathways.

Damage to prefrontal areas is known to impair the inhibitory control of spontaneous and reactive behaviours. In the realm of eye movements, prefrontal lesions cause difficulties in suppressing the visual grasp reflex as evident from increased error rates in antisaccade tasks [33, 34, 35]. In keeping with the notion of a prefrontal dysfunction in ALS, increased error rates during antisaccade and delayed saccade tasks have also been observed in ALS patients [29, 30, 3]. In addition, Shaunak et al. [30] report an elevated frequency of SWJ in ALS in comparison to controls, in apparent agreement with the patient's deficient inhibitory control. However, a later study only observed an increase in SI amplitude, but found the fixation time between SI events to be similar in ALS and controls [29], suggesting that there were no changes in SI frequency. Finally, Gorges et al. [36] who evaluated the rate of eye displacement (sum of vectorial SI amplitudes per unit of time) rather than amplitudes and frequencies per se, found a 1.4-fold increase of this rate in ALS. This value is difficult to reconcile with the almost four-fold increase in SWJ frequency reported by Shaunak et al. [30] whereas it would seem to be compatible with the unaltered SI frequency and the moderate increase in amplitude of Donaghy et al. [29].

In view of these contradictory reports we wished to reappraise whether or not ALS patients exhibit an increased frequency of SI. The demonstration of an elevated frequency would constitute circumstantial evidence that SI result from involuntary oculomotor noise that is minimised by inhibitory control in healthy individuals but released in patients. Moreover, we were interested to learn which other characteristics of SI might be affected in ALS, such as the percentage of particular patterns (SWJ, stair case, etc) or the preferred SI direction; changes of direction have been observed in Alzheimer's Disease and Mild Cognitive Impairment [37]. Finally, we also wanted to establish which of those oculomotor tasks that are known to reflect prefrontal functions (antisaccades, delayed saccades) would best correlate with the ALS-related changes of SI, and likewise for the neuro-psychological scores testing the cognitive status of ALS patients.

## Methods

### Participants

The present study is based on the oculomotor and neuropsychological data of 119 ALS patients (mean age 61.0, 25%-quantile 52.3, median 61.6, 75%-quantile 70.9; 64 male, 55 female) and on the oculomotor data of a cohort of 47 age-matched control subjects without any history of neurological or psychiatric diseases (mean age 61.4, 25%-quantile 54.0, median 63.9, 75%-quantile 70.9; 23 male, 24 female). The patients' data which are harboured by the German Motor Neuron Disease network (MND-Net) represent the results of standardised oculomotor and neuropsychological tests administered routinely as part of the patients' general clinical work-up in our department. The data of controls were obtained using exactly the same oculomotor test protocol as with patients. The study had been approved by the ethics committee of the University of Ulm (references #19/2012 and #20/2012) and subjects had given their written consent.

### Equipment

Participants were seated in a comfortable chair at the centre of a white hemicylindrical screen with an eye-to-screen distance of 1.50 m and were donned with a head-mounted video-oculography system (**VOG**, EyeSeeCam ^®^ ) approved for medical purposes according to DIN EN ISO 14971. The screen carried arrays of red and green light emitting diodes (**LED**s; invisible when not lit) spaced 5° along the horizontal circumference and the vertical meridian of the screen. The LEDs subtended 0.3° of visual angle and were used to elicit reactive refixation saccades. Smooth pursuit eye movements could be elicited by projecting a light spot onto the screen via horizontally and vertically moving mirror galvanometers. The VOG system sampled the movements of both eyes at a frequency of 220 Hz, displayed them on-line to the examiner and transferred them to Matlab ^®^ files.

### Procedures

After adjusting the VOG cameras, the room lighting was dimmed to a standardised low-illumination level, and participants were presented with sinusoidal movements of the light spot in the horizontal plane (frequency 0.125 Hz, amplitude ±20°) followed by vertical movements (±15°) which elicited slow smooth pursuit movements serving calibration. Thereafter, participants were to steadily fixate at the central LED for 30 s. The present report is based on the intrusions encountered during this period. After the fixation period, first horizontal and then vertical refixation saccades were elicited by randomly lit LEDs (not considered here). Finally, three tasks testing executive functions were presented: (1) rapidly alternating voluntary saccades (**RAVS**), (2) delayed saccades and (3) antisaccades. In the RAVS condition, participants were to saccade back and forth for 30 s as frequently as they could between two permanently lit LEDs first spaced apart 40° horizontally (**RAVSX**) and then 30° vertically (**RAVSY**). The delayed saccade task required participants to withhold their automatic reaction to the appearance of a new target ('visual grasp reflex') and to continue fixation at the current target until an acoustic “go” command was sounded after a randomly varying delay of 1.5 to 2.5 s (16 trials; horizontal steps from old to new target ranging from 5° to 40° in pseudorandom order). In the antisaccade task, participants fixated at the central red LED until it disappeared and a green LED was lit at 5, 10, 15 or 20° to the left or the right, respectively (16 trials in pseudorandom order). Participants then were to immediately saccade into the opposite empty hemifield and to try to fixate at the (imagined) mirror position of the green target until a red LED was lit at this position after a pseudorandomly varying delay of 2.4 to 3 s. For RAVS, the number of saccades achieved within 30 s was scored, for delayed saccades the percent number of premature reactions, and for antisaccades the percent number of errors (first saccade directed at the green target).

The cognitive status was assessed in 91 of the 119 ALS patients by an experienced neuropsychologist using the German version of the Edinburgh Cognitive and Behavioural ALS screen (ECAS) [38]. ECAS consists of 15 individual tasks evaluating ALS specific cognitive changes (language, verbal fluency, executive functions including social cognition) as well as non-specific ones (memory and visuo-spatial abilities). Finally, all patients were clinically assessed in terms of the revised form of the ALS functional rating scale (ALSFRS-R) [39].

### Data Analysis

**Figure 1.**
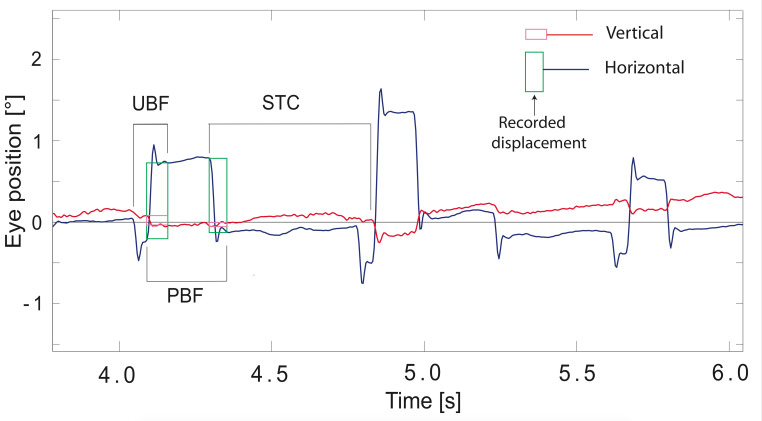
Example of saccadic intrusions recorded in an ALS patient. Blue trace shows horizontal eye position, red vertical. Rectangles framing the leftmost intrusions illustrate the results of manual analysis (green for horizontal, magenta for vertical component); their height captures the *de facto* eye displacement caused by the intrusion. Acronyms above and below pairs of successive intrusions illus-trate the definition of the patterns **STC** (stair case), **UBF** (asymmetrical back-and forth) and **PBF** (symmetrical back-and-forth).

Data analysis was carried out with dedicated in-house Matlab ^®^ programmes and Excel ^®^ macros. The recordings of horizontal and vertical eye position from the two eyes were orthogonalised and calibrated on the basis of the horizontal and vertical pursuit responses, converted into cyclopean signals by an averaging procedure, and displayed on a computer screen. Intrusions were identified by visual inspection based on their characteristic pattern featuring conspicuous dynamic overshoots (see Fig. 1) and [19] followed in some cases by damped oscillations (own observation). Using the larger of the two eye movement components (in the vast majority the horizontal one), the evaluator positioned two vertical hair lines, one at the intrusion's onset and one at the end of the dynamic overshoot processes so as to capture the *de facto* displacement produced by the intrusion (see Fig. 1). The horizontal and vertical eye displacements during the epoch marked by the two hairline positions were stored in files together with the SI onset time for further processing. In addition, we noted the number of eye blinks occurring during the fixation period (detection based on the characteristic VOG-artefact caused by blinks).

In a first step, the Cartesian notation of eye displacement was converted into a vector representation using polar co-ordinates, and the distribution of the vector amplitudes of each participant was plotted for a coarse survey using a logarithmic binning. The vector amplitudes then were sorted according to vector orientation into one of eight direction sectors of ±22.5° each, centred on the cardinal directions. Moreover, each vector was assigned to one of four patterns by comparing it to its predecessor in time (see Fig.1): (1) stair case (**STC**) if the intrusion had the same direction as the preceding one; (2) back-and-forth (**BAF**) if the intrusion had opposite direction; (3) paired back-and-forth (**PBF**), a subdivision of BAF comprising only cases where the directional planes of the two saccades differed by less than 22.5° and their amplitude ratio stayed inside the range 0.8 to 1/0.8; (4) unpaired BAF (**UBF**) not meeting these criteria. As evident from Fig. 1, each intrusion, except the very first and last ones, occurred twice in the course of pattern assignment, once as the trailing component of a first pattern and then as the leading component of the following pattern. The leading component determined the pattern direction, whereas the pattern amplitude was equated to the amplitude of the trailing component (cf. Discussion) or, in the case of PBF, to the average of both components. PBF intrusions are actually analogues of the classical SWJ without the a priori interval duration constraints used by automated detection algorithms [e.g. 40].

For each participant, the following descriptive parameters were calculated separately for each of the 8 x 4 combinations of direction sectors and patterns: average and median values of the vectorial amplitude (**VAMP**) and of the interval (**INTV**) between the leading and trailing component of the pattern, the displacement rate (**DISR**) calculated as the sum of VAMP per unit of time (because VAMP is always positive, the sum increases monotonously during the fixation period), and the frequency of occurrence (**FREQ**) of the pattern. From these individual data, sample averages representative of the ALS and control populations, respectively, were calculated, again separately for all directions and patterns. Ultimately, the results were visualised in the form of polar graphs (Fig. 3).

**Figure 2.**
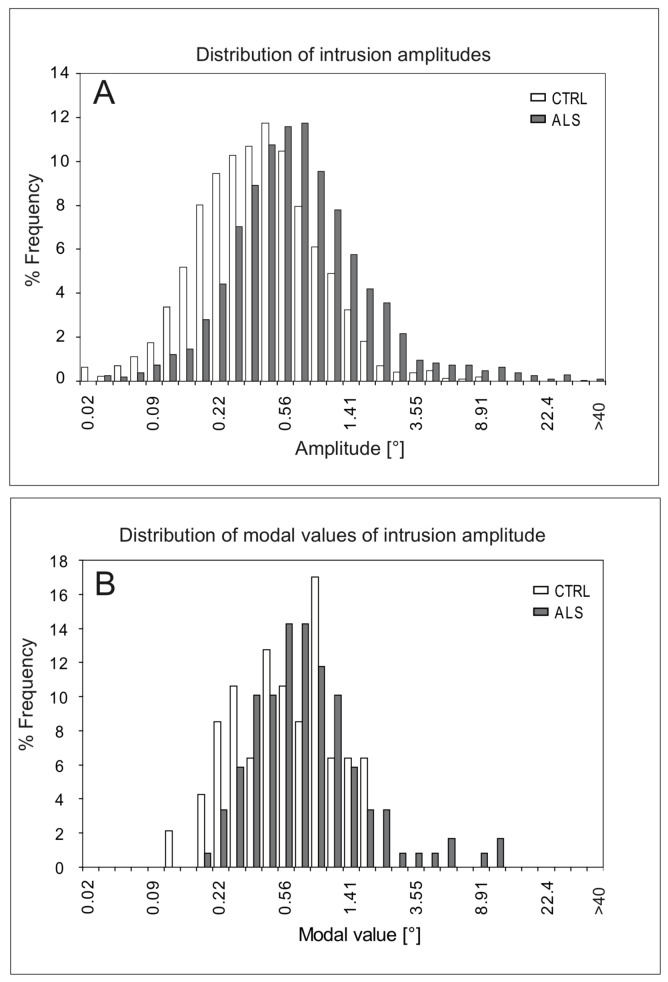
Frequency distribution of intrusion amplitudes in ALS and controls (CTRL). **A**, amplitudes of intrusions forming BAF patterns, pooled data from all participants; N= 5132 (ALS) and N=2206 (CTRL). **B**, modal values of subjects’ individual amplitude distributions (intrusions belonging to STC and BAF patterns pooled); N=47 (CTRL) and N=119 (ALS). Note logarithmic scaling of bins in both panels.

**Figure 3.**
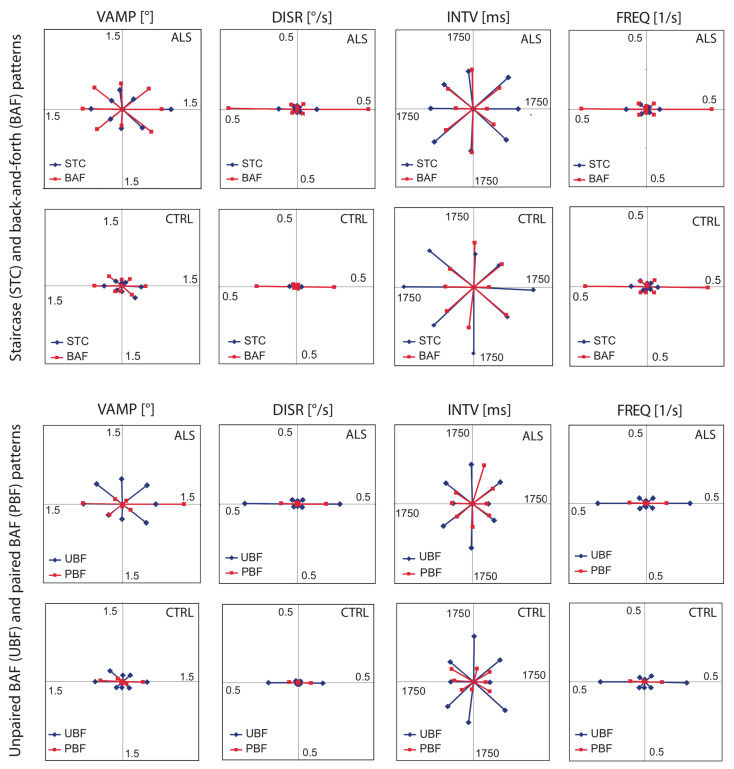
Vectorial representation of intrusion parameters of ALS patients (1 ^st^ and 3 ^rd^ row of panels) and controls (2 ^nd^ and 4 ^th^ row). Upper half of figure shows results from stair case (**STC**, blue symbols) and back-and-forth (**BAF**, red) patterns; lower half, separate analysis of unpaired (**UBF**, blue) and paired (**PBF**, red) BAF intrusions, respectively. 1 ^st^ column of panels,** **VAMP** (vectorial amplitude, average of individual medians); 2 ^nd^ , **DISR** (displacement rate, average of individual sums of vectorial amplitudes per unit of time); 3 ^rd^ , **INTV** (interval between first and second component of pattern, average of individual medians); 4 ^th^ , **FREQ** (frequency of occurrence, average of individual frequencies). Vector orientations reflect the average direction of the intrusions within each sector of ±22.5°.

### Statistics

Statistic inferences were based exclusively on non-parametric tests. For comparisons within groups (ALS, controls), the Wilcoxon test for matched pairs was used. Between groups, the Mann-Whitney U-test was applied. To test relationships between parameters, Spearman's rank correlation coefficient was calculated. In view of the interdependence of many variables and the many possible comparisons we mostly refrain from claiming significance but report only the uncorrected 2-sided percent error probabilities as recommended by Perneger [41].

## Results

### Qualitative observations

Fig. 2a illustrates the distributions of the vectorial amplitude VAMP in ALS patients and controls, respectively, based on all intrusions contributing to BAF patterns which in both groups account for more than 70% of all SI (ALS, gray bars, N=5132; controls, white, N=2206). Whereas Fig. 2a is biased in favour of individuals producing many SI, Fig. 2b provides an unbiased summary in terms the modal values of each participant's amplitude distribution. In both histograms, the ALS distributions were shifted toward larger amplitude values in comparison to controls, suggesting hat the intrusions of ALS were larger on average. The distribution of the modal values (Fig. 2b) alerts to a considerable interindividual variability in both groups and demonstrates that also a number of ALS patients produced intrusions predominantly in the microsaccade range (<0.25°).

A more detailed view of the results is provided by Fig. 3 which shows polar representations of the sample averages of VAMP and INTV (both based on the participants' median values), and of DISR and FREQ, all sorted according to vector direction, intrusion pattern and group. The results illustrated in Fig. 3 suggest that there were many *qualitative* similarities between ALS and controls (error probabilities in parentheses hold for horizontal intrusions):

Considering first STC and BAF intrusions, we note from the corresponding FREQ diagrams in the upper two rows of panels that in both groups

(1) the majority of intrusions was confined to the horizontal plane (ALS 70%, Controls 67%),

(2) the percentages of intrusions in the vertical (ALS 5%, controls 6%) and each of the oblique planes (ALS 12 and 13%, controls 13 and 14%) were virtually identical in both groups, and

(3) BAF intrusions occurred more frequently than STC intrusions (both groups p < 10 ^-5^ ).

The INTV diagrams indicate that in both groups horizontal BAF stood out by the shortness of their intervals when compared to non-horizontal ones (ALS; p < 10 ^-5^ ; controls, p < 0.007) and to STC in all directions.

The synopsis of the VAMP, DISR and FREQ, diagrams indicates that

(1) in both groups the displacement rates of non-horizontal BAF and STC were negligible although within each pattern the amplitudes often were of roughly the same order of magnitude in all directions. Thus, the displacement rate (which reflects the product of amplitude and frequency) was essentially determined by the pattern frequency. By the same token,

(2) the displacement rates of STC were much smaller than those of BAF (both groups p < 10 ^-5^ ).

Also after separating BAF into UBF and PBF, many *qualitative* similarities were observed between ALS and controls (see lower two rows of panels):

(1) UBF intrusions occurred more frequently than PBF (controls 80 vs 20%, ALS 78 vs 22%; both groups p < 10 ^-5^ ) and produced larger displacement rates, therefore (both groups p < 10 ^-5^ ).

(2) The intervals of horizontal UBF and PBF were nearly identical.

(3) Paired BAF intrusions occurred almost exclusively in the horizontal plane.

However, besides these many qualitative similarities between ALS and controls, one *qualitative difference* between the two groups can be suspected: There was a right-left asymmetry of PBF intrusions in ALS with rightward VAMP, DISR, and FREQ being larger than leftward ones (p = 9·10 ^-4^ , 2·10 ^-5^ , and 2·10 ^-4^ , respectively), whereas no such asymmetry was seen in controls (p≥ 0.1 all parameters). Otherwise, no consistent left-right asymmetries were observed.

### Quantitative comparison between ALS and controls

The results of the *quantitative* comparison between ALS and controls are listed in Tables 1 and 2 in terms of sample averages, medians and error probabilities. Noticeably, with regard to SI frequency, no difference between the two groups could be demonstrated. Actually, the frequencies obtained in the two groups were almost identical (Table 1, FREQ). Apart from that, the *quantitative* comparison between ALS and controls confirmed that ALS made larger SI than controls and exhibited correspondingly larger displacement rates (Table 1, VAMP and DISR), except for paired BAF intrusions to the left. This exception reflects the directional asymmetry of PBF mentioned above which appears to penalise the left direction at the profit of rightward SI.

**Table 1:** Parameters of saccadic intrusions in ALS-patients and controls. Data based on horizontally oriented intrusions only.

			STC		BAF		UBF		PBF	
			R	L	R	L	R	L	R	L
VAMP	ALS	Avg	1.18	0.74	1.12	1.02	0.96	1.03	1.38	0.82
		*Med*	*0.47*	*0.44*	*0.70*	*0.64*	*0.67*	*0.62*	*0.65*	*0.49*
	CTRL	Avg	0.40	0.36	0.54	0.57	0.54	0.58	0.41	0.46
		*Med*	*0.25*	*0.27*	*0.43*	*0.47*	*0.41*	*0.47*	*0.35*	*0.36*
		p	0.003	0.004	0.002	0.000	0.003	0.000	0.000	0.054
DISR	ALS	Avg	0.12	0.12	0.45	0.44	0.27	0.34	0.18	0.10
		*Med*	*0.04*	*0.04*	*0.31*	*0.28*	*0.19*	*0.21*	*0.08*	*0.04*
	CTRL	Avg	0.03	0.04	0.24	0.25	0.16	0.20	0.08	0.06
		*Med*	*0.02*	*0.02*	*0.13*	*0.15*	*0.10*	*0.12*	*0.02*	*0.03*
		p	0.008	0.007	0.004	0.000	0.005	0.005	0.001	> 0.1
INTV	ALS	Avg	1113	1052	458	537	488	545	378	378
		*Med*	*923*	*842*	*416*	*459*	*430*	*452*	*305*	*305*
	CTRL	Avg	1318	1576	438	790	457	646	365	490
		*Med*	*743*	*927*	*358*	*469*	*363*	*484*	*279*	*381*
		p	> 0.1	0.031	0.039	> 0.1	0.020	> 0.1	> 0.1	> 0.1
FREQ	ALS	Avg	0.08	0.13	0.42	0.41	0.28	0.31	0.13	0.11
		*Med*	*0.06*	*0.09*	*0.41*	*0.39*	*0.27*	*0.30*	*0.09*	*0.06*
	CTRL	Avg	0.07	0.10	0.39	0.38	0.29	0.29	0.11	0.09
		*Med*	*0.03*	*0.12*	*0.36*	*0.33*	*0.24*	*0.25*	*0.06*	*0.09*
		p	> 0.1	> 0.1	> 0.1	> 0.1	> 0.1	> 0.1	> 0.1	> 0.1

**CTRL, **controls; **Avg,** sample averages; **Med**, sample medians; **VAMP**, vector amplitude [°]; **DISR**, displacement rate [°/s]; **INTV**, interval duration [ms]; **FREQ**, frequency [1/s]; **STC**, stair case pattern; **BAF**, back-and-forth pattern; **UBF**, unpaired BAF pattern; **PBF**, paired BAF pattern; **p**, uncorrected two-sided error probabilities of the Mann-Whitney U-test comparing the sample averages of controls and ALS patients.

Other quantitative differences between ALS and controls comprise elevated eye blink rates, larger percent errors in the delayed and antisaccade tasks and a reduced number of rapidly alternating voluntary saccades in ALS (Table 2).

**Table 2:** Oculomotor executive scores and frequency of eye lid blinks in ALS patients and controls.

	ALS		CTRL		
	N	Avg	N	Avg	p
Blinks	119	0.25	47	0.13	< 10 ^ -4 ^
RAVSX	118	1.54	43	1.77	0.0040
RAVSY	117	1.58	40	1.82	0.0022
E_DSX	116	33	42	10	<10 ^ -5 ^
E_ASX	114	46	41	25	<10 ^ -4 ^

**CTRL**, controls;** N**, number of individuals; **Avg**, score averages; **p**, uncorrected two-sided error probabilities (Mann-Whitney U-tests) comparing ALS to controls; **RAVSX** ( **RAVSY**), frequency of rapidly alternating voluntary saccades in horizontal (vertical) direction [1/s]; **E_DSX** ( **E_ASX),** percent error rate in delayed (antisaccade) test.

### Correlation with executive functions

The rank correlation coefficients obtained in ALS between the amplitudes, displacement rates and frequencies of horizontal SI on the one hand, and a number of executive parameters on the other hand, are visualised by the matrices in Fig. 4. From left to right the matrices correspond to the patients' median VAMP, average DISR, and FREQ. The correlations were calculated separately for each of the four patterns (STC, BAF, UBF, PBF) and for each the two directions. Colours code the error probabilities, and the sign symbols indicate positive and negative co-variations. We note several points from these matrices:

(1) In all cases of an error probability of less than 0.05 (coloured cells), the signs of the co-variations in a given row were identical in all three matrices.

(2) In all cases of an error probability of less than 0.05, the correlation coefficients indicated an increase of VAMP, DISR, and FREQ when the executive parameters reflected a functional worsening. For example, a decrease of the number of rapidly alternating voluntary saccades, an increase of erroneous responses in the delayed saccade task and a reduced ECAS fluency score (EC_Flun) all reflect an impaired performance. Accordingly, there was a negative co-variation of all SI parameters with RAVS and EC_Flun, and a positive one with the delayed saccade error rate (E_DSX).

(3) Across SI parameters the closest co-variation occurred with the delayed saccade error rate which also was the only score to exhibit a possibly trustworthy relationship with the frequency of intrusions.

(4) Among the ECAS scores, verbal fluency (EC_Flun) and the compound score EC_Spec were the best related ones to SI parameters, whereas the visuo-spatial score (EC_Visp) exhibited a conspicuous lack of co-variation with VAMP in contrast to all other parameters.

(5) For PBF intrusions (the analogues of SWJ) the direction appears to matter since the number correlations with low error probability was clearly larger for rightward as compared to leftward SI, paralleling the right dominance of PBF amplitudes and displacement rates in ALS.

(6) The eye blink rate correlated positively with SI amplitude and in particular so with that of BAF intrusions

(7) The functional ALS rating scale (ALSFRS-R, FRS in Fig. 4) exhibited little co-variation with SI parameters.

In contrast to ALS, the intrusions of controls exhibited no signs of co-variation with the executive oculomotor parameters except for a possible co-variation of the frequency of PBF intrusions with the delayed saccade errors and with the number RAVS. Finally, neither in ALS nor in controls was there any correlation between the intrusion parameters and participants' age.

## Discussion

We have studied saccadic intrusions (SI) in ALS patients and controls during their attempt to steadily fixate at a central fixation point in the absence of other stimuli or attention absorbing tasks. The intrusions of ALS patient proved to be an up-scaled version of those exhibited by controls with larger amplitudes but otherwise similar characteristics (preferred direction, proportion of square wave jerks). In particular, SI frequency did not differ between the two groups. In ALS, the amplitudes of SI increased along with the worsening of performance in oculomotor and neuropsychological tests of executive functions whereas controls exhibited little or no such co-variations.

Fig. 4 Correlation of saccadic intrusion parameters with oculomotor and neuropsychologic scores in ALS patients. The matrices represent the sign and significance level of Spearman's rank correlation coefficients of (1) the vectorial amplitude (**VAMP, **individual median values), (2) the displacement rate **(DISR**) and (3) the frequency (**FREQ**) of horizontal intrusions with the following nine scores: (1) Number of eye lid **Blinks** during the fixation period; (2) **RAVSX**, number of saccades in the horizontal RAVS task; (3) **RAVSY**, vertical analogue of RAVSX; (4) **E_DSX**, percent errors in the horizontal delayed saccade task; (5) **E_ASX**, percent errors in the horizontal antisaccade task; (6) **EC_Visp**, visuospatial ECAS-score; (7) **EC_Flun**, verbal fluency ECAS-score; (8) **EC_Spec** compound ALS-specific ECAS-score; (9) **FRS**, revised ALS-functional rating scale (ALSFRS-R). In each matrix, intrusions are subdivided into stair case movements (**STC**), back and forth movements (**BAF**), unpaired BAF (**UBF**) and paired BAF (**PBF**) as well as into rightward and leftward. Colours code the uncorrected two-sided error probabilites (white, p≥0.05; yellow, 0.01≤p<0.05; orange, 0.001≤p<0.01; red, p<0.001), the plus- and minus signs indicate positive and negative co-variation, respectively.

We first will comment on the definition of intrusion patterns as used here and by other authors. Based on a comparison of the SI characteristics of our controls with previous reports we will argue that our methods produced results that are in line with those obtained with more elaborated procedures. We then will consider those features of saccadic intrusions which are qualitatively similar in ALS and controls, before dealing with the differences between the two groups and with the co-variation between intrusions and other oculomotor and neuropsychological parameters. This will lead us to the question of how the ALS patients' deficit of inhibitory control can lead to larger SI amplitudes without changing SI frequency.

### Pattern definition

Our pattern definition differs from previous classifications of SI in that it ignores whether a given SI departs from, or returns to, the target position. Because routine recordings in a clinical environment do not always allow for an exact determination of this position at the scale of microsaccades, we considered instead the *relative* direction of each SI with respect to the *physical* direction of the preceding SI. Hence, all SWJ are PBF patterns, but not all PBF patterns represent SWJ in the 'classical' sense. In fact, according to our definition, the saccades framing the interval between two consecutive SWJ of similar amplitude and direction form, in combination with this between-SWJ interval, another PBF pattern. Because between-SWJ intervals are generally longer than typical within-SWJ intervals, the median interval durations of PBF patterns of both ALS and controls (Table 1) clearly exceeded the 'ideal' 200 ms SWJ interval assumed by Otero-Millan et al. [40] and the range of about 250 to 300 ms reported for healthy individuals. One might ask whether the lack of a constraint on the intervals (e.g. interval ≤ 250 ms) inherent to our analysis disguised the increase in frequency of SWJ reported by Shaunak et al. [30]. If the percentage of paired BAF patterns with intervals <250 ms (i.e. of SWJ) was small in controls but large in ALS, the lack of an interval constraint would indeed hide an increase of SWJ. If that were the case, the intervals of paired BAF patterns would be longer in controls than in ALS. However, the median intervals of ALS and controls were similar with both paired and unpaired BAF (Mann-Whitney U-test, p>0.5) thus giving no hint at different proportions of SWJ in ALS and controls. Moreover, even if interval duration mattered, this would not touch our main finding that, in spite of the patients' difficulty to suppress inappropriate behaviours, the frequency of their SI was not larger than in controls, nor would it invalidate the observed qualitative similarities and differences between ALS and controls.

Our equating the amplitude of patterns to that of their trailing component may appear inconsistent given that the pattern direction is determined by the leading component. We used this description because we had expected that STC patterns would mostly occur as part of small corrections and would be identified as such by small amplitudes. As evident from Table 1 and Fig. 3, this expectation was not borne out. STC patterns, like the one marked in Fig. 1, mostly occurred as part of a change in direction of successive BAF patterns and had similar amplitudes as BAF, therefore.

### Saccadic intrusions in controls

In controls, the sample averages of the individual median vectorial amplitudes ranged from 0.36 to 0.58° across patterns, and the global distribution which cumulates results from all participants peaked at 0.45° (Fig 1a). These values compare favourably with reported averages which mostly had values in the order of 0.4 to 0.6° [23, 27, 42] and with published distributions which had peaks ranging from 0,3 to 0,5° [23, 28, 29, 37]. Since the global distribution conceals interindividual differences, we constructed also the frequency distribution of the individual modal amplitude values (Fig. 1b) which revealed a considerable variability among subjects. The 90% quantile of this distribution corresponded to individual modal amplitudes spanning a range of about four octaves in ALS and three in controls. In about 4% of the ALS patients and 14% of the controls the modal amplitudes were within the microsaccade range (VAMP < 0.25° [5]).

The frequency of occurrence clearly varied across the four patterns, being lowest with STC and PBF, and largest with BAF. The reason for the low STC frequency is obvious: There are rarely more than three consecutive intrusions of similar direction since otherwise large fixation errors would accumulate. Moreover, the standard situation leading to a STC is a change in direction of successive BAF. However, BAF often occur as a series of isodirectional volleys leaving no chance for STC. Pooling STC and BAF frequencies, controls produced intrusions at a rate of 0.47/s in total, which is within the range of published values (0.3/s [23] to 1.8/s [22]). The frequency of PBF-intrusions, like that of SWJ, obviously depends on the constraints defining these patterns. Previous classification criteria for SWJ varied from visual identification (e.g. [21]) to elaborated algorithms [27]. Although our definition of the PBF pattern did not constrain the duration of the interval, and although we accepted as PBF also the pattern formed by the saccades framing the interval between successive SWJ of similar size and direction (cf. above), our value of 0.1/s fits into the low end of the range of published SWJ-frequencies (0.04/s [26] to 0.45 [18]).

In summary, the present data are compatible with previous work on SI and add, as new aspects, the distribution of individual modal values and an analysis of stair case intrusions. Also, they invalidate concerns that the use of non-punctual, albeit small, targets could have been inappropriate. The 0.3° of visual angle used here (required by the clinical environment) is actually about the upper limit beyond which SI become appreciably larger [43, 44].

### Qualitatively common features of ALS and controls

The relatively high and very similar frequency figures (about 30%) of non-horizontal SI in both groups should not blind to the fact that of the eight orientation sectors considered, the horizontal ones exhibited by far the largest SI frequency and displacement rates (Fig. 3). A horizontal predominance has been repeatedly demonstrated in controls [23, 27, 40]. Small deviations from this horizontal predominance have been reported in cases of Alzheimer's Disease and of Mild Cognitive Impairment [37]. Although our analysis used an only coarse binning of the intrusion directions, a systematic inclination of the preferred plane would have rotated the vectors representing the 0° and 180° sectors out of the horizontal plane, but no such rotation was detected. Similar as reported in the above studies, there were only few predominantly vertical intrusions, and a number of these were probably corrections for spontaneous up- or downward drifts of the eyes rather than true SI. The tightest sticking to the horizontal plane with virtually no vertical examples was seen with PBF intrusions, in agreement with the data of Otero-Millan [27]. Thus, there were no qualitative differences between ALS and controls in the form of different direction preferences (for the one exception in the form of a rightward bias of paired BAF intrusions in ALS see below).

### Differences between ALS and controls: an effect of impaired inhibitory control?

The observed elevated error rates of ALS patients in the delayed and antisaccade tasks is consistent with previous reports on ALS [29, 30, 36] and also in line with studies of other neurodegenerative diseases such as PSP [45], Alzheimer’s disease [46] and fronto-temporal dementia (FTD) [46, 47] that overlap with ALS clinically, genetically and pathologically [48, 49]. A common denominator here is the affection of frontal and prefrontal areas involved in inhibiting automated reflexive behaviours. With regard to eye movements, the dorsolateral prefrontal cortex, the frontal eye fields and the supplementary eye fields are of particular importance [50]. The reduced number of rapidly alternating voluntary saccades of our ALS patients indicates that, besides the suppression of inappropriate eye movements, also the pacing of voluntary actions is impaired in ALS. The reduction may be related to a dysfunction of the supplementary eye fields which are supposed to play a role in the timing of saccades [51]. Taken together, all tests of executive oculomotor functions support the notion of a prefrontal dysfunction in our ALS patients.

A main goal of the present study was to reappraise whether intrusions are more frequent in ALS patients than in controls. Our results provide no evidence for such an increase of SI frequency. This result concurs with the observation of Donaghy et al. [29] that ALS patients exhibit similar intervals between SI occurrences and, hence, probably similar frequencies. We cannot confirm the almost fourfold increase of SWJ frequency in ALS reported by Shaunak et al. [30]. Each of the four intrusion patterns occurred with almost the same frequency in our ALS patients and in controls. In particular, also the frequency of PBF intrusions, the closest analogue of SWJ, did not increase in ALS. A possible reason for the discrepancy between our results and those of Shaunak et al. [30] could be the fact that their analysis was restricted to saccades exceeding 0.5°, a value close to the modal value of the amplitude distribution in controls. Because the distribution of ALS patients is shifted toward larger values, the number of SI recorded in their patients must have been larger in comparison to controls thus feigning an elevated frequency.

As already noted above, there can be little doubt that our ALS patients' inhibitory control was impaired. They made significantly more errors in both the antisaccade and delayed saccade tasks. These tasks clearly test the ability to suppress compulsory reflexive behaviours. Moreover, unlike Byrne [52] we also observed a clear increase of eye blinks in ALS. Elevated blink rates likewise point to a lack of inhibitory control after damage to prefrontal regions [53, 54].

The observed association of the above indicators of impaired inhibitory control with the increase of SI amplitude in ALS (Fig. 4) and the simultaneous lack of a corresponding increase of SI frequency is puzzling. If one adopts the view that SI are, by and large, involuntary noisy events, one would expect that the patients' impaired inhibitory control causes an increase of SI frequency in ALS. In other neurodegenerative disorders associated with prefrontal dysfunctions (PSP, MSA) such an increase has indeed been observed in addition to the enlargement of the amplitudes [27]. In contrast to these findings, the only weak hint at an elevated distractibility that can be derived from our ALS patients' SI might be the conspicuous left-right asymmetry of their PBF amplitudes which did not occur in controls. This could be an example of uncovering an undesired covert attention (55) attracted by the presence of the examiner on the participants' right side. In support of this conjecture, the frequency of rightward PBF intrusions was the only pattern frequency to exhibit a possibly trustworthy correlation with the error rate of delayed saccades.

On the other hand, if one ascribes a purposeful role to SI, it is difficult to understand why a dysfunction of inhibitory mechanisms should increase their amplitudes. Is this increase then necessary to maintain the hypothetical function of SI under the conditions of ALS, or does it result from the malfunction of frontal cortical mechanisms that control other aspects of subcortical eye movement generation unrelated to inhibition?

Clearly, an explanation of how SI frequency can remain unaffected while SI amplitude increases is difficult without understanding why, where and how SI are generated. At present there is no generally accepted response to this question although the frontal eye fields [56], the basal ganglia [57, 58] and the rostral superior colliculus [59, 60] have been implied. Evidence from studies in monkey indicates that an inactivation of these structures can indeed lead to dissociated changes of SI amplitude and frequency. For example, reversible cooling of the simian frontal eye fields increased SI amplitude while the frequency remained constant or even decreased (results from the pre-cue epoch of a delayed saccade experiment, [56]). Likewise, inactivation of rostral sites of the simian superior colliculus by muscimol injection resulted in a broader amplitude distribution of SI and a reduction of SI frequency, at least if a non-punctual fixation object (4° of visual angle) was presented [60]. Although the superior colliculus itself becomes only weakly affected during late stages of ALS (Del Tredici-Braak, personal communication) its functioning is likely to be altered indirectly when upstream structures become deficient (frontal cortex, striatal area of basal ganglia). Thus, in principle at least, a frontal and/or basal ganglia dysfunction may have dissociated effects on SI amplitude and frequency. Finally, since the amplitude of saccades but not their timing is under control of the cerebellar fastigial nucleus [61], one could envision a dysfunction of the cerebellar amplitude control in the aftermath of the affection of the precerebellar nuclei which constitute a gateway to the cerebellum; these nuclei, but not the cerebellum itself, become affected during ALS-stage 2 [32]. However, goal-directed saccades of ALS patients exhibit no signs of hypermetria [30, 36]. Therefore, it is difficult to ascribe the isolated increase of SI amplitude in ALS to a cerebellar dysfunction.

To better understand the differential behaviour of SI amplitude and SI frequency in ALS, future research may try to compare the SI of ALS patients and controls in situations of competition between the fixation task and simultaneous attentional demands in order to assess whether the SI frequency of ALS will be modulated to a larger extent than that of controls as one would expect in view of their prefrontal dysfunction. Also a detailed comparison of the pathologic brain alterations in ALS to those in neurodegenerative diseases exhibiting a parallel increase of SI amplitude and frequency may provide further insight.

Although the central population tendencies of the SI amplitudes differed significantly between controls and ALS patients (cf. Table 1), the considerable overlap of the amplitudes at the level of individual subjects (cf. Fig. 2) precludes a contribution of SI to the diagnosis of ALS. However, one could explore whether low or large SI amplitudes in diagnosed cases of ALS point to some specific subtype or genetic form of the disease or are related to the individual time course of disease progression so as to allow a distinction between 'slow' and 'fast progressors'. Moreover, combined with other oculomotor phenotypes (executive error rates, signs of impaired oculomotor brain stem functions) SI amplitude might help to characterize the state of ALS in terms of oculomotor functions at the individual level ('oculomotor staging' [36]).

### Correlation with ECAS scores and oculomotor executive scores

Of all individual ECAS scores, verbal fluency was the one to show the best correlation with the patients' SI amplitude. This result fits with the notion that fluency appears to be a particularly sensitive indicator of cognitive alterations in ALS [62]. The compound score (EC_Spec), which summarises the outcome of the nine individual tests of ALS-specific alterations, exhibited a qualitatively similar but weaker correlation with SI amplitude. Notably, both scores exhibited almost no co-variation with SI frequency. In contrast, the score of the patients' visuo-spatial abilities (EC_Visp), which is an ALS-unspecific score, did not co-vary with SI amplitude. Taken together, these results demonstrate a parallel increase of the SI amplitudes and the worsening of ALS-specific cognitive scores.

Among the oculomotor executive scores the error rate of delayed saccades was a better predictor of SI alterations than that of antisaccades. This is a somewhat surprising result since the delayed saccade task requires only a suppression of the initial visual grasping reflex whereas correct antisaccades require in addition to reflex suppression a mirror transformation of the target coordinates and the generation of a voluntary response to the imagined target. The reason for this weaker co-variation could be the small variation range of the antisaccade error rate which is due to the already elevated baseline value of 25% suggested by the performance of controls. Rapidly alternating voluntary saccades exhibited the weakest co-variation with SI amplitude and displacement rate in ALS, in agreement with the relatively small difference of this score between ALS and controls. Since RAVS would seem to rely little on inhibitory control, this observation concurs with the conjecture that the elevated SI amplitudes of ALS reflect in a specific, still to be elucidated way the patients' decline of inhibitory control.

### Conclusion

During the attempt to fixate at a stationary target in the absence of other attention requiring tasks, ALS patients made larger but, surprisingly, not more frequent saccadic intrusions than control subjects although tests of their executive functions suggested an impairment of inhibitory prefrontal mechanisms. Taken in isolation, this result does not support the view that intrusions are noisy events that are minimised by inhibitory control. Yet, studies in other neurodegenerative diseases (e.g. PSP, MSA), which report more frequent SI as a correlate of prefrontal malfunction, would seem to favour such a view.

Although studies in monkey point to the possibility that changes of saccade amplitude and frequency can be dissociated during malfunction of areas involved in saccade generation, these findings in animal cannot be directly transferred to the current results in diseased humans.

Future studies should address different oculomotor phenotypes of altered fixation control, in particular a comparison of ALS with neurodegenerative diseases that are associated with an increase of SI frequency. That way, the analysis of oculomotor functions including attempted steady fixation might be established as one approach to gain insights into higher functional networks and their specific disorder-associated malfunctions, and thereby to characterise the neuropsychological status of patients.

## Ethics and Conflict of Interest

The authors declare that the contents of the article are in agreement with the ethics described in http://biblio.unibe.ch/portale/elibrary/BOP/jemr/ethics.html and that there is no conflict of interest regarding the publication of this paper. 

## Acknowledgement

We are indebted to Dr. R. Jürgens for writing the Matlab programmes and to Prof. H. Braak and Dr. Dr. K. Del Tredici-Braak for a helpful discussion.

## References

[b23] Abadi, R. , & Gowen, E. ( 2004, 10). Characteristics of saccadic intrusions. Vision Research, 44, 2675–2690. 10.1016/j.visres.2004.05.009 0042-6989 15358063

[b19] Abadi, R. , Scallan, C. J. , & Clement, R. A. ( 2000). The characteristics of dynamic overshoot in square-wave jerks, and in congenital and manifest latent nystagmus. Vision Research, 40, 2813–2829. 10.1016/S0042-6989(00)00146-2 0042-6989 10960653

[b62] Abrahams, S. , Leigh, P. N. , Harvey, A. , Vythelingum, G. N. , Grise, D. , & Goldstein, L. H. ( 2000). Verbal fluency and executive dysfunction in amyotrophic lateral sclerosis (ALS). Neuropsychologia, 38, 734–747. 10.1016/S0028-3932(99)00146-3 0028-3932 10689049

[b49] Al-Chalabi, A. , van den Berg, L. H. , & Veldink, J. (2017).Gene discovery in amyotrophic lateral sclerosis: Implications for clinical management. Nature Reviews. Neurology,13,96–104. 10.1038/nrneurol.2016.182 1759-4758 27982040

[b25] Alexander, R. G. , Macknik, S. L. , & Martinez-Conde, S. (2018).Microsaccade Characteristics in Neurological and Ophthalmic Disease. Frontiers in Neurology,9,144. 10.3389/fneur.2018.00144 1664-2295 29593642 PMC5859063

[b53] Anagnostou, E. , Kouzi, I. , Vassilopoulou, S. , Paraskevas, G. P. , & Spengos,K. (2012).Spontaneous eyeblink rate in focal cerebrovascular lesions. European Neurology,67,39–44. 10.1159/000333063 0014-3022 22156766

[b57] Averbuch-Heller,L. , Stahl,J. S. , Hlavin,M. L. , & Leigh,R. J. (1999,1 1).Square-wave jerks induced by pallidotomy in parkinsonian patients. Neurology,52,185–188. 10.1212/WNL.52.1.185 0028-3878 9921873

[b13] Bahill,A. T. , Clark,M. R. , & Stark,L. (1975).The main sequence, a tool for studying human eye movements. Mathematical Biosciences,24,191–204. 10.1016/0025-5564(75)90075-9 0025-5564

[b1] Barlow,H. B. (1952).Eye movements during fixation. The Journal of Physiology,116,290–306. 10.1113/jphysiol.1952.sp004706 0022-3751 14939180 PMC1392140

[b47] Boxer,A. L. , Garbutt,S. , Seeley,W. W. , Jafari,A. , Heuer,H. W. , Mirsky,J. , Hellmuth,J. , Trojanowski,J. Q. , Huang,E. , DeArmond,S. ,. . .. (2012,4).Saccade abnormalities in autopsy-confirmed frontotemporal lobar degeneration and Alzheimer disease. Archives of Neurology,69,509–517. 10.1001/archneurol.2011.1021 0003-9942 22491196 PMC3423186

[b32] Braak,H. , Brettschneider,J. , Ludolph,A. C. , Lee,V. M. , Trojanowski,J. Q. , & Del,T. K. (2013,12).Amyotrophic lateral sclerosis—A model of corticofugal axonal spread. Nature Reviews. Neurology,9,708–714. 10.1038/nrneurol.2013.221 1759-4758 24217521 PMC3943211

[b48] Brettschneider,J. , Del,T. K. , Irwin,D. J. , Grossman,M. , Robinson,J. L. , Toledo,J. B. , Fang,L. , Van,D. V. , Ludolph,A. C. , Lee,V. M. ,. . .. (2014,3).Sequential distribution of pTDP-43 pathology in behavioral variant frontotemporal dementia (bvFTD). Acta Neuropathologica,127,423–439. 10.1007/s00401-013-1238-y 0001-6322 24407427 PMC3971993

[b52] Byrne,S. , Pradhan,F. , Ni,D. S. , Treacy,M. , Cassidy,L. , & Hardiman,O. (2013,5).Blink rate in ALS. Amyotrophic Lateral Sclerosis & Frontotemporal Degeneration,14,291–293. 10.3109/21678421.2012.729217 2167-8421 23151260

[b39] Cedarbaum,J. M. , Stambler,N. , Malta,E. , Fuller,C. , Hilt,D. , Thurmond,B. , & Nakanishi,A. (1999,10 31).The ALSFRS-R: A revised ALS functional rating scale that incorporates assessments of respiratory function. BDNF ALS Study Group (Phase III). Journal of the Neurological Sciences,169,13–21. 10.1016/S0022-510X(99)00210-5 0022-510X 10540002

[b45] Chen,A. L. , Riley,D. E. , King,S. A. , Joshi,A. C. , Serra,A. , Liao,K. , Cohen,M. L. , Otero-Millan,J. , Martinez-Conde,S. , Strupp,M. , & Leigh,R. J. (2010).The disturbance of gaze in progressive supranuclear palsy: Implications for pathogenesis. Frontiers in Neurology,1,147. 10.3389/fneur.2010.00147 1664-2295 21188269 PMC3008928

[b5] Collewijn,H. , & Kowler,E. (2008).The significance of microsaccades for vision and oculomotor control. Journal of Vision (Charlottesville, Va.),8,20–21. 10.1167/8.14.20 1534-7362 PMC3522523 19146321

[b54] Colzato,L. S. , van den Wildenberg,W. P. , van Wouwe,N. C. , Pannebakker,M. M. , & Hommel,B. (2009,7).Dopamine and inhibitory action control: Evidence from spontaneous eye blink rates. Experimental Brain Research,196,467–474. 10.1007/s00221-009-1862-x 0014-4819 19484465 PMC2700244

[b20] Daroff,R. B. (1977,1).Ocular oscillations. The Annals of Otology, Rhinology, and Laryngology,86,102–107. 10.1177/000348947708600118 0003-4894 835966

[b7] Ditchburn,R. W. (1980).Letter to the editors The function of small saccades. Vision Research,20,271–272. 10.1016/0042-6989(80)90112-1 0042-6989 7385601

[b6] Ditchburn,R. W. , Fender,D. H. , & Mayne,S. (1959).Vision with controlled movements of the retinal image. The Journal of Physiology,145,98–107. 10.1113/jphysiol.1959.sp006130 0022-3751 13621424 PMC1356943

[b2] Ditchburn,R. W. , & Ginsborg,B. L. (1953).Involuntary eye movements during fixation. The Journal of Physiology,119,1–17. 10.1113/jphysiol.1953.sp004824 0022-3751 13035713 PMC1393034

[b29] Donaghy,C. , Pinnock,R. , Abrahams,S. , Cardwell,C. , Hardiman,O. , Patterson,V. , McGivern,R. C. , & Gibson,J. M. (2009,3).Ocular fixation instabilities in motor neurone disease. A marker of frontal lobe dysfunction? Journal of Neurology,256,420–426. 10.1007/s00415-009-0109-x 0340-5354 19306041

[b24] Doslak,M. J. , Dell’Osso,L. F. , & Daroff,R. B. (1983).Multiple double saccadic pulses occurring with other saccadic intrusions and oscillations. Neuro-Ophthalmology (Aeolus Press),3,109–116. 10.3109/01658108309009726 0165-8107

[b55] Engbert,R. , & Kliegl,R. (2003,4).Microsaccades uncover the orientation of covert attention. Vision Research,43,1035–1045. 10.1016/S0042-6989(03)00084-1 0042-6989 12676246

[b8] Engbert,R. (2006).Microsaccades: A microcosm for research on oculomotor control, attention, and visual perception. Progress in Brain Research,154,177–192. 10.1016/S0079-6123(06)54009-9 0079-6123 17010710

[b46] Garbutt,S. , Matlin,A. , Hellmuth,J. , Schenk,A. K. , Johnson,J. K. , Rosen,H. , Dean,D. , Kramer,J. , Neuhaus,J. , Miller,B. L. , Lisberger,S. G. , & Boxer,A. L. (2008,5).Oculomotor function in frontotemporal lobar degeneration, related disorders and Alzheimer’s disease. Brain,131,1268–1281. 10.1093/brain/awn047 0006-8950 18362099 PMC2367697

[b60] Goffart,L. , Hafed,Z. M. , & Krauzlis,R. J. (2012,8 1).Visual fixation as equilibrium: Evidence from superior colliculus inactivation. The Journal of Neuroscience : The Official Journal of the Society for Neuroscience,32,10627–10636. 10.1523/JNEUROSCI.0696-12.2012 0270-6474 22855812 PMC3473086

[b36] Gorges,M. , Müller,H. P. , Lulé,D. , Del,T. K. , Brettschneider,J. , Keller,J. , Pfandl,K. , Ludolph,A. C. , Kassubek,J. , & Pinkhardt,E. H. (2015).Eye Movement Deficits Are Consistent with a Staging Model of pTDP-43 Pathology in Amyotrophic Lateral Sclerosis. PLoS One,10,e0142546. 10.1371/journal.pone.0142546 1932-6203 26559944 PMC4641606

[b16] Gowen,E. , Abadi,R. V. , & Poliakoff,E. (2005,12).Paying attention to saccadic intrusions. Brain Research. Cognitive Brain Research,25,810–825. 10.1016/j.cogbrainres.2005.09.002 0926-6410 16256318

[b61] Guerrasio,L. , Quinet,J. , Büttner,U. , & Goffart,L. (2010,4).Fastigial oculomotor region and the control of foveation during fixation. Journal of Neurophysiology,103,1988–2001. 10.1152/jn.00771.2009 0022-3077 20130038

[b33] Guitton,D. , Buchtel,H. A. , & Douglas,R. M. (1985).Frontal lobe lesions in man cause difficulties in suppressing reflexive glances and in generating goal-directed saccades. Experimental Brain Research,58,455–472. 10.1007/BF00235863 0014-4819 4007089

[b17] Hafed,Z. M. , & Clark,J. J. (2002,10).Microsaccades as an overt measure of covert attention shifts. Vision Research,42,2533–2545. 10.1016/S0042-6989(02)00263-8 0042-6989 12445847

[b59] Hafed,Z. M. , Goffart,L. , & Krauzlis,R. J. (2009,2 13).A neural mechanism for microsaccade generation in the primate superior colliculus. Science,323,940–943. 10.1126/science.1166112 0036-8075 19213919 PMC2655118

[b18] Herishanu,Y. , & Sharpe,J. A. (1981).Normal square wave jerks. Investigative Ophthalmology & Visual Science,20,268–272.0146-0404 7461930

[b21] Jung,R. , & Kornhuber,H. H. (1964).Results of electronystagmography in man: the value of optokinetic, vestibular and spontaneous nystagmus for neurologic diagnosis and research. In M. B. Bender (Ed.), The Oculomotor System (pp. 428–482).Hoeber Medical Division, Harper & Row.

[b37] Kapoula,Z. , Yang,Q. , Otero-Millan,J. , Xiao,S. , Macknik,S. L. , Lang,A. , Verny,M. , & Martinez-Conde,S. (2014,4).Distinctive features of microsaccades in Alzheimer’s disease and in mild cognitive impairment. Age (Dordrecht, Netherlands),36,535–543. 10.1007/s11357-013-9582-3 2452-0756 PMC4039256 24037325

[b31] Kiernan,M. C. , Vucic,S. , Cheah,B. C. , Turner,M. R. , Eisen,A. , Hardiman,O. , Burrell,J. R. , & Zoing,M. C. (2011,3 12).Amyotrophic lateral sclerosis. Lancet,377,942–955. 10.1016/S0140-6736(10)61156-7 0140-6736 21296405

[b15] Krauzlis,R. J. , Goffart,L. , & Hafed,Z. M. (2017,4 19).Neuronal control of fixation and fixational eye movements. Philosophical Transactions of the Royal Society of London. Series B, Biological Sciences,372,372. 10.1098/rstb.2016.0205 0962-8436 PMC5332863 28242738

[b51] Kunimatsu,J. , & Tanaka,M. (2012,11).Alteration of the timing of self-initiated but not reactive saccades by electrical stimulation in the supplementary eye field. The European Journal of Neuroscience,36,3258–3268. 10.1111/j.1460-9568.2012.08242.x 0953-816X 22845785

[b38] Lulé,D. , Burkhardt,C. , Abdulla,S. , Böhm,S. , Kollewe,K. , Uttner,I. , Abrahams,S. , Bak,T. H. , Petri,S. , Weber,M. , & Ludolph,A. C. (2015,3).The Edinburgh Cognitive and Behavioural Amyotrophic Lateral Sclerosis Screen: A cross-sectional comparison of established screening tools in a German-Swiss population. Amyotrophic Lateral Sclerosis & Frontotemporal Degeneration,16,16–23. 10.3109/21678421.2014.959451 2167-8421 25292386

[b34] Machado,L. , & Rafal,R. D. (2004,5).Control of fixation and saccades during an anti-saccade task: An investigation in humans with chronic lesions of oculomotor cortex. Experimental Brain Research,156,55–63. 10.1007/s00221-003-1765-1 0014-4819 14685809

[b9] Martinez-Conde,S. , Macknik,S. L. , & Hubel,D. H. (2004,3).The role of fixational eye movements in visual perception. Nature Reviews. Neuroscience,5,229–240. 10.1038/nrn1348 1471-003X 14976522

[b12] Martinez-Conde,S. , Macknik,S. L. , Troncoso,X. G. , & Hubel,D. H. (2009,9).Microsaccades: A neurophysiological analysis. Trends in Neurosciences,32,463–475. 10.1016/j.tins.2009.05.006 0166-2236 19716186

[b10] Martinez-Conde,S. , Otero-Millan,J. , & Macknik,S. L. (2013,2).The impact of microsaccades on vision: Towards a unified theory of saccadic function. Nature Reviews. Neuroscience,14,83–96. 10.1038/nrn3405 1471-003X 23329159

[b43] McCamy,M. B. , Najafian,J. A. , Otero-Millan,J. , Macknik,S. L. , & Martinez-Conde,S. (2013).The effects of fixation target size and luminance on microsaccades and square-wave jerks. PeerJ,1,e9. 10.7717/peerj.9 2167-8359 23638403 PMC3628898

[b42] McGivern,R. C. , & Gibson,J. M. (2006).Characterisation of ocular fixation in humans by analysis of saccadic intrusions and fixation periods: A pragmatic approach. Vision Research,46,3741–3747. 10.1016/j.visres.2006.05.016 0042-6989 16889812

[b44] Ohtsuka,K. , Mukuno,K. , Ukai,K. , & Ishikawa,S. (1986).The origin of square wave jerks: Conditions of fixation and microsaccades. Japanese Journal of Ophthalmology,30,209–215.0021-5155 3761744

[b27] Otero-Millan,J. , Serra,A. , Leigh,R. J. , Troncoso,X. G. , Macknik,S. L. , & Martinez-Conde,S. (2011,3 23).Distinctive features of saccadic intrusions and microsaccades in progressive supranuclear palsy. The Journal of Neuroscience : The Official Journal of the Society for Neuroscience,31,4379–4387. 10.1523/JNEUROSCI.2600-10.2011 0270-6474 21430139 PMC3111217

[b40] Otero-Millan,J. , Schneider,R. , Leigh,R. J. , Macknik,S. L. , & Martinez-Conde,S. (2013).Saccades during Attempted Fixation in Parkinsonian Disorders and Recessive Ataxia: From Microsaccades to Square-Wave Jerks. PLoS One,8,e58535. 10.1371/journal.pone.0058535 1932-6203 23516502 PMC3596296

[b56] Peel,T. R. , Hafed,Z. M. , Dash,S. , Lomber,S. G. , & Corneil,B. D. (2016,8).A Causal Role for the Cortical Frontal Eye Fields in Microsaccade Deployment. PLoS Biology,14,e1002531. 10.1371/journal.pbio.1002531 1544-9173 27509130 PMC4980061

[b41] Perneger,T. V. (1998).What’s wrong with Bonferroni adjustements. BMJ (Clinical Research Ed.),316,1236–1238. 10.1136/bmj.316.7139.1236 0959-8138 PMC1112991 9553006

[b50] Pierrot-Deseilligny,C. , Müri,R. M. , Ploner,C. J. , Gaymard,B. , & Rivaud-Pechoux,S. (2003).Cortical control of ocular saccades in humans: A model for motricity. In C. Prablanc , D. Pélisson , & Y. Rossetti (Eds.), Progress in Brain research:142.Elsevier Science B.V (pp. 2–16). 10.1016/S0079-6123(03)42003-7 12693251

[b28] Pinnock,R. A. , McGivern,R. C. , Forbes,R. , & Gibson,J. M. (2010).An exploration of ocular fixation in Parkinson’s disease, multiple system atrophy and progressive supranuclear palsy. Journal of Neurology,257,533–539. 10.1007/s00415-009-5356-3 0340-5354 19847469

[b35] Ploner,C. J. , Gaymard,B. M. , Rivaud-Pechoux,S. , & Pierrot-Deseilligny,C. (2005,5 15).The prefrontal substrate of reflexive saccade inhibition in humans. Biological Psychiatry,57,1159–1165. 10.1016/j.biopsych.2005.02.017 0006-3223 15866556

[b11] Poletti,M. , & Rucci,M. (2010,3 24).Eye movements under various conditions of image fading. Journal of Vision (Charlottesville, Va.),10,6–18. 10.1167/10.3.6 1534-7362 PMC2951333 20377283

[b26] Rascol,O. , Sabatini,U. , Simonetta-Moreau,M. , Montastruc,J.-L. , & Clanet,M. (1991).Square wave jerks in Parkinsonian syndromes. Journal of Neurology, Neurosurgery, and Psychiatry,54,599–602. 10.1136/jnnp.54.7.599 0022-3050 PMC1014429 1895124

[b3] Ratliff,F. , & Riggs,L. (1950).Involuntary motions of the eye during monocular fixation. Journal of Experimental Psychology,40,687–701. 10.1037/h0057754 0022-1015 14803643

[b58] Shaikh,A. G. , Xu-Wilson,M. , Grill,S. , & Zee,D. S. (2011,5).‘Staircase’ square-wave jerks in early Parkinson’s disease. The British Journal of Ophthalmology,95,705–709. 10.1136/bjo.2010.179630 0007-1161 20693560

[b22] Shallo-Hoffmann,J. , Petersen,J. , & Mühlendyck,H. (1989).How normal are “normal” square wave jerks? Investigative Ophthalmology & Visual Science,30,1009–1011.0146-0404 2722436

[b30] Shaunak,S. , Orrell,R. W. , O’Sullivan,E. , Hawken,M. B. , Lane,R. J. M. , Henderson,L. , & Kennard,C. (1995).Oculomotor function in amyotrophic lateral sclerosis: Evidence for frontal impairment. Annals of Neurology,38,38–44. 10.1002/ana.410380109 0364-5134 7611722

[b4] Yarbus,A. L. (1967).Eye Movements and Vision. (English Translation).Plenum Press. 10.1007/978-1-4899-5379-7

[b14] Zuber,B. L. , Stark,L. , & Cook,G. (1965).Microsaccades and the velocity-amplitude relationship for saccadic eye movements. Science,150,1459–1460. 10.1126/science.150.3702.1459 0036-8075 5855207

